# Effects of Ramadan fasting on cardiovascular risk factors: a prospective observational study

**DOI:** 10.1186/1475-2891-11-69

**Published:** 2012-09-10

**Authors:** Mohsen Nematy, Maryam Alinezhad-Namaghi, Masoud Mahdavi Rashed, Mostafa Mozhdehifard, Seyedeh Sania Sajjadi, Saeed Akhlaghi, Maryam Sabery, Seyed Amir R Mohajeri, Neda Shalaey, Mohsen Moohebati, Abdolreza Norouzy

**Affiliations:** 1 Department of Nutrition, Mashad, Biochemistry and Nutrition, Endoscopic & Minimally Invasive Surgery, and Cancer Research Centers, University of Medical Sciences, Mashad, 91779-48564, Iran; 2 Biochemistry and Nutrition Research Center and Department of Nutrition, Mashad, University of Medical Sciences, Mashad 91779-48564, Iran; 3 Department of Community Medicine, Mashad, University of Medical Sciences, Mashad, 91779-48564, Iran; 4 Iranian Applied Research Center for Health and Sustainable Development (IRCPHD), North Khorasan University of Medical Sciences, North Khorasan, Iran; 5 Department of Cardiology, Ghaem Teaching Hospital, Mashad, University of Medical Sciences, Mashad, 91766-99199, Iran

**Keywords:** Ramadan, Fasting, hs-CRP, Homocysteine, FBS, Insulin resistance, Cardiovascular diseases, Risk factors

## Abstract

**Background:**

Previous research has shown that Ramadan fasting has beneficial effects on cardiovascular risk factors, however there are controversies. In the present study, the effect of Ramadan fasting on cardiovascular risk factors has been investigated.

**Method:**

This is a prospective observational study that was carried out in a group of patients with at least one cardiovascular risk factor (including history of documented previous history of either coronary artery disease (CAD), metabolic syndrome or cerebro-vascular disease in past 10 y). Eighty two volunteers including 38 male and 44 female, aged 29–70 y, mean 54.0 ± 10 y, with a previous history of either coronary artery disease, metabolic syndrome or cerebro-vascular disease were recruited. Subjects attended the metabolic unit after at least 10 h fasting, before and after Ramadan who were been fasting for at least 10 days. A fasting blood sample was obtained, blood pressure was measured and body mass index (BMI) was calculated. Lipids profile, fasting blood sugar (FBS) and insulin, homocysteine (hcy), high-sensitivity C-reactive protein (hs-CRP) and complete blood count (CBC) were analyzed on all blood samples.

**Results:**

A significant improvement in 10 years coronary heart disease risk (based on Framingham risk score) was found (13.0 ± 8 before Ramadan and 10.8 ±7 after Ramadan*, P <*0.001, *t* test).There was a significant higher HDL-c, WBC, RBC and platelet count (PLT), and lower plasma cholesterol, triglycerides, LDL-c, VLDL-c, systolic blood pressure, body mass index and waist circumference after Ramadan (*P <*0.05, *t* test). The changes in FBS, insulin,Homeostasis Model Assessment Insulin Resistance (HOMA-IR), hcy, hs-CRP and diastolic blood pressure before and after Ramadan were not significant (*P >*0.05, *t* test).

**Conclusions:**

This study shows a significant improvement in 10 years coronary heart disease risk score and other cardiovascular risk factors such as lipids profile, systolic blood pressure, weight, BMI and waist circumference in subjects with a previous history of cardiovascular disease.

## Introduction

Ramadan is a month for Muslims during which they abstain from eating, drinking and smoking from dawn to sunset.

During month of Ramadan there are changes in the quality of food and eating patterns. It might be due to the consumption of more carbohydrates and sweet foods, mainly in the form of two large meals at dawn and sunset [1]. It has been established that a given nutrient ingested at an unusual time can induce different metabolic effects [2]; however, the physiological changes in Ramadan are not well known.

Cardiovascular diseases are the leading cause of death in the world [3]. Fasting during Ramadan is essentially a radical change in lifestyle for the period of one lunar month that may affect cardiovascular risk in patients with coronary artery disease (CAD) and cerebro-vascular disease (CVD) [4,5].

The most common risk factors associated with increased risk of atherosclerotic heart disease or stroke are abnormalities in plasma lipids and some coagulation and haemostatic factors, hypertension and smoking [6].

Lipids profile are affected by factors such as changes in the dietary habits, using different dietary fats, increased consumption of refined sugar, and reduced physical activity [7].

Many studies reported a significant reduction in type 2 diabetic patients' weight during Ramadan [8,9], some other reported a non-significant reduction in weight of these patients [10]. Bouguerra et al. showed Ramadan fasting has a moderate effect on glycaemia and lipoprotein levels in type 2 diabetic patients when previous metabolic control was quite good; but fasting induced more deterioration when previous control was poor [9].Although, another study in Iran showed a deterioration of glycaemic control after Ramadan fasting in Type 2 diabetic patients that was more evident in patients using oral hypoglycaemic medication than diet-controlled patients [11]. Shariatpanahi et al. showed that the combined change in the number and timing of meals and portioning of the entire intake into only two meals per day may increase insulin sensitivity in Ramadan [8]. Homocysteine (Hcy) is a well-known mixed amino acid, intermediary on the metabolic pathway between methionine and cysteine [12]. Mild to moderate elevation of plasma hcy concentration has been known as carrying an increased risk for the development of atherosclerotic vascular disease [13]. Hyperhomocysteinemia is thus an independent risk factor for atherosclerosis, and impaired arterial endothelial function is detectable in healthy adults with hyperhomocysteinaemia [13].

High-sensitivity C-reactive protein (hs-CRP), a plasma inflammation marker, has been known to play a role in the development of cardiovascular diseases and is considered as a biomarker that is useful in the prediction of early cardiovascular risk [14]. Several prospective epidemiological studies have demonstrated a consistent relationship between higher C-reactive protein (CRP) levels and increased risk of cardiovascular events, including myocardial infarction, stroke, and cardiovascular death [15,16]. CRP may reflect a greater burden of atherosclerosis or alternatively may identify a high-risk atherosclerosis phenotype with an active inflammation and atherosclerotic plaque that is vulnerable to rupture [17,18].

Fasting is not meant to create excessive hardship for individuals who are not able to do that, the Islamic practice exempts many people from fasting, including those who are ill and fasting is harmful for them [19]. One of the common questions for patients with a history of cardiovascular disease is whether it is safe for them to fast during Ramadan. Yet, studies on the effects of Ramadan fasting on blood lipids, blood pressure, anthropometric parameters and other cardiovascular risk factors are scarce, and have given inconclusive results [9,10,20].

In current research, the effect of Ramadan fasting on cardiovascular risk factors including biochemical indices, blood pressure and main anthropometric parameters has been investigated.

## Subjects and methods

### Subjects

This was a prospective observational study that was carried out in a group of patientswith at least one cardiovascular risk factor (including history of documented previous history of either coronary artery disease (CAD), metabolic syndrome or cerebro-vascular disease in past 10 y). Subjects' risk factors were compared before and after Ramadan month to see the effects of fasting on it.

This study was conducted according to the guidelines laid down in the Declaration of Helsinki and all procedures involving human subjects were approved by the Research Ethics Committee of Mashad University of Medical Sciences (approval number 87224). Written informed consent was obtained from all subjects. This study was conducted in the month of Ramadan during September/October 2008 in city of Mashad, Iran.

Volunteers were recruited from outpatient cardiology clinics in Imam Reza and Ghaem teaching hospitals via poster advertisement or direct invitation. Inclusion criteria were male and female between 29 and 70 years old with a history of documented previous history of either coronary artery disease (CAD), metabolic syndrome or cerebro-vascular disease in past 10 y.76 patients were taking medications including 52 patients on antihypertensive agents,47 patients onstatins, 26patients on oral antidiabetic medications including metformin and sulphonyleureas and 46 patients on antiplatelet aggregation agents. Six patients did not take any medications.

Diagnosis of cardiovascular disease was based of cardiologist judgment.

Exclusion criteria were pregnant and lactating women, subjects with the age of below 18 y old and those who fasted for less than 10 d. Subjects were nonsmoker and volunteers with any type of acute inflammation (hs-CRP >5) were excluded. One hundred and one subjects participated in the first part of the study; however, 82 subjects completed the study. Seventeen patients were excludedbecause of fasting for less than 10 d and 2 subjects withdrew their consent for personal reasons.

Past medical history, drug and smoking history were obtained prior the study.

### Blood sampling and data gathering

Subjects attended the metabolic unit after 10–12 h fasting, in two stages; between 7 d before and 2 first days of starting Ramadan and from the 27^th^ of Ramadan till 6 d after end of this month. An 8 mL fasting blood sample was collected from the median cubital vein by using vacuum sampling method. The blood samples were analyzed in an University affiliated laboratory.

Systolic and diastolic blood pressures were measured in duplicate by sphygmomanometers (Omron M3, M4, Omron Corporation, the Netherlands). If difference between two measurements was more than 5%, a third reading was performed and average of two near readings was considered as mean blood pressure. Food frequency questionnaire (FFQ) was completed in both stages before and after Ramadan at the same Ramadan in the matched BMI from the control group.

Anthropometric parameters such as height, body weight and waist circumference were measured in both phases. Body mass index (BMI) was calculated as body weight (kg) divided by squared height in meters (m^2^). Height and weight were measured, using standardized procedures. Height was measured using a portable stadiometer (OTM, Tehran, Iran). Height measurement was taken to the nearest 0.1 cm, without shoes, with the subject stretching to the maximum height and the head positioned in the Frankfort plane. Weight was measured using Rassa weight scale (Rassa, Tehran, Iran) to the nearest 100 g with subjects removing shoes and in light clothing. Waist circumference was measured to the nearest 0.1 cm at the mid-point between the lower rib and the upper margin of the iliac crest [21] in a horizontal plane by using a no stretching tape with an insertion buckle at one end.

### Assays

Lipids profile, fasting blood sugar (FBS), fasting insulin, hs-CRP, hcy and complete blood count (CBC) were analyzed using standard methods. Homocysteine measurement was being performed on blood plasma and the other variables on blood serum.

Triglyceride (TG) and cholesterol were measured byGPO-PAP and CHOD-PAP methods respectively (Parsazmun kit, Karaj, Iran, for both). The direct method was used for determiningthe HDL and LDL by Human kits (Human Gesellschaft fur Biochemica und Diagnostica GmbH, Max-Planck-Ring 21 – D-65205 Wiesbaden – Germany) and fasting blood glucose was measured by glucose-oxidize, GOD-PAP method [22](Parsazmun kit, Karaj, Iran). Total hcy measurement was performed with immunoturbidometry, based on assays to co-substrate conversion product [23,24] (Demeditec Diagnostic GmbH, Lise-Meitner-strabe 2, D-24145 Kiel Germany). Insulin was measured, using radioimmunoassay methods (ELISA) with IRMAkit (Immunotecha.s. Prague, Czech Republic). Hs-CRP concentration was measured by immunoturbidometry method by auto analyzer [25] (Prestige 24i, Tokyo Boeki, Japan). Insulin resistance was estimated from the homeostasis model assessment of insulin resistance (HOMA-IR) originally described by Matthews et al. [26] as calculated from the following equation:

(1) $$\left[\text{Fasting insulin concentration}\left(µ\text{U}/\text{mL}\right)\times \text{fasting glucose concentration}\left(\text{mmol}/\text{L}\right)\right]/22.5$$

[22] Cell blood counts were measured by KX-21 N Sysmexautoanalyser system (Sysmex Shanghai Ltd, Lujiazu, China).

Framingham coronary heart disease risk score was used to calculate the risk of CAD [27].

### Data analysis

The Kolmogorov-Smirnov test was performed to assess the normal distribution. Differences between results were analyzed, using paired samples *t* test for normally distributed parameters and Wilcoxon Signed Ranks test for not normally distributed parameters (LDL, TG, FBS, HOMA-IR and hs-CRP). Relationship between dichotomous and quantitative variables was assessed by independent sample *t*test and correlation between quantitative and quantitative variables was evaluated by Pearson’s correlation. Two-wayrepeated measures ANOVA were used to assess interactions among variables seem to be effective on parameter differences (*P <*0.15 in Pearson’s correlation or independent sample *t* test) on variables before and after Ramadan. Quantitative data were expressed as the mean ± SD for all parameters. Statistical significance was considered at *P <*0.05 for all tests. Relationship among cholesterol serum level and existing CAD, using statin family medication and hypertension (based on history) were evaluated by using two-way repeated measures ANOVA. These statistical analyses were conducted by using SPSS statistical software (version 11.5, SPSS Inc. Chicago, IL).

## Results

Duration of daily fasting during Ramadan 2008 was between 14 h and 42 minin first day and 13 h and 35 min in last day in Mashad, Iran. The mean days subjects fasted in this study was 26.3 ± 5 (range 10–35 d).

Eighty two volunteers including 38 male and 44 female, age 29–70 y, mean 54.0 ± 10 y, with a previous history of either coronary artery disease (CAD) (60.9%), metabolic syndrome (67%) or cerebro-vascular disease (2.4%) in past 10 y completed the study. Twenty eight percent of them (23 volunteers) were suffering from both CAD and metabolic syndrome.

### 10 years coronary heart disease risk

This study shows a significant improvementin 10 years coronary heart disease risk, based on Framingham risk score (13.0 ± 8 before Ramadan and 10.8 ±7 after Ramadan*, P <*0.001, *t* test).

### Lipids profile

The values of total cholesterol, triglycerides, VLDL-c, LDL-c, cholesterol/HDL and LDL/HDL ratio were significantly decreased (*P* = 0.02 for cholesterol and *P <*0.001 for rest, Wilcoxon test for triglycerides and LDL, and paired *t* test for rest) and HDL-c increased significantly (*P <*0.001, paired *t* test) after Ramadan fasting (Table1).

**Table 1:** Effect of Ramadan fasting on lipids profile before and after Ramadan (n = 82)

***Biochemical variables***	***Before Ramadan***	***After Ramadan***	***p-values***
	$\bar{x}\pm SD^{3}$	$\bar{x}\pm SD^{3}$	
***Cholesterol***(mg/dL)	193.4 ± 51	184.3 ± 42	0.023
***Triglycerides*** (mg/dL)	224.8 ± 129	182.9 ± 112	0.000
***HDL-c*** ( mg/dL )	43.0 ± 9	47.9 ± 8	0.000
***Cholesterol/HDL***(ratio)	4.5 ± 1	3.9 ± 1	0.000
***VLDL-c*** (mg/dL )	38.1 ± 14	33.8 ± 17	0.000
***LDL-c*** (mg/dL )	109.96 ± 46	96.83 ±35	0.000
***LDL/HDL*** (ratio)	2.5 ± 0.8	2.0 ± 0.6	0.000

^***3***^SD = standard deviation.

It appears that there was no significant effects of CAD,hypertension and taking statin drugs on cholesterol mean level variations before and after Ramadan (*P* = 0.12, *P* = 0.09 and *P* = 0.71 respectively). Effect of Ramadan fasting on cholesterol was independent of existing CAD, hypertension and taking statins (*P* = 0.04, ANOVA).

### Blood pressure

There was a significant decrease in systolic blood pressure (132.9 ± 16 mmHg,129.9 ± 17 mmHg, *P* = 0.03, paired *t* test). Although, no significant change in diastolic blood pressure was found (80.2 ± 9, 78.6 ± 11 mmHg, *P* = 0.14, paired *t* test).

### FBS, insulin, HOMA-IR, homocysteine, hs-CRP

As it is shown in Table2, FBS, insulin, HOMA-IR, hcy and hs-CRP concentrations showed no significant difference after Ramadan fasting (*P* = 0.33, *P* = 0.58, *P* = 0.76, *P* = 0.06 and *P* = 0.07 respectively, paired *t* test for insulin and hcy and Wilcoxon test for rest).

**Table 2:** Effect of Ramadan fasting on some clinical and biological parameters before and after Ramadan (n = 65 for hs-CRP and 82 for rest)

***Biochemical variables***	***Before Ramadan***	***After Ramadan***	***p-values***
	$\bar{x}\pm SD^{3}$	$\bar{x}\pm SD^{3}$	
***FBS***^***1***^(mmol/l)	124.7 ± 48	128.1 ± 54	0.327
***insulin*** (μU/mL)	12.4 ± 9.5	11.8 ± 7.6	0.583
***HOMA.IR*** (μUmol/l^2^)	3.8 ± 3	3.6 ± 3	0.093
***Hcy***^***2***^(μmol/l)	10.5 ± 3.8	11.5 ± 3.9	0.065
***hs-CRP*** ( mg/L)	1.0 ± 1	0.9 ± 1	0.160

^***1***^*FBS*, fasting blood sugar.

^***2***^Hcy, homocysteine.

^***3***^SD = standard deviation.

### Anthropometric parameters

Body weight, BMI and waist circumference reduced significantly after Ramadan fasting (73.5 ± 13, 71.7 ± 13 kg, 28.4 ± 4, 27.7 ± 4 kg/m^2^ and98.3 ± 9, 96.7 ± 9 cm respectively and *P <*0.001, paired *t* test).

### Hematological parameters

Table3 illustrates the values regarding hematological parameters before and after Ramadan. The mean concentrations of all these parameters (except hemoglobin and Hematocrit) showed significant changes when compared before and after Ramadan (*P* = 0.25 and *P* = 0.32 respectively, paired *t* test). There was a significant higher WBC, RBC, platelet count (PLT) (*P* = 0.01, *P <*0.001, *P <*0.001 respectively, paired *t* test), and significant lower MCV and MCH (*P <*0.001, paired *t* test) after Ramadan.

**Table 3:** Effect of Ramadan fasting on hematological factors before and after Ramadan (Mean values with standard deviation) (n = 82)

***Hematological factors***	***Before Ramadan***	***After Ramadan***	***p-values***
	$\bar{x}\pm SD^{3}$	$\bar{x}\pm SD^{3}$	
***WBC*** (1000/μl)	6.6 ± 1.5	6.9 ± 1.5	0.011
***RBC*** (mil/μl)	4.7 ± 0.3	4.9 ± 0.4	0.000
***HGB***^***1***^(g/dL)	13.6 ± 1.2	13.7 ± 1.0	0.254
***MCV*** (fl)	87.6 ± 5.0	83.7 ± 4.2	0.000
***MCH*** (pg)	28.8 ± 2.1	27.9 ± 1.8	0.000
***PLT***^***2***^ (1000/μl)	219.2 ± 54.1	242.7 ± 56.2	0.000

^*1*^*HGB,* Hemoglobin.

^*2*^*PLT,*Platelet count.

^***3***^SD = standard deviation.

There was no significant correlation among number of fasting days and studied parameters in this study.

### Food records

FFQ results are shown in Table4. The data show a statistically non-significant reduction in energy and other macronutrients intake during fasting in month of Ramadan.

**Table 4:** Effect of Ramadan fasting on energy and nutrient intake of control group before and after Ramadan (Mean values with standarddeviation) (n =82)

***Consumption***	***Before Ramadan***	***After Ramadan***	***p-values***
	$\bar{x}\pm SD^{1}$	$\bar{x}\pm SD^{1}$	
***Energy (kcal)***	2588.6 ±1485	2449.3 ± 1179	0.337
***Carbohydrates(g/day)***	327.8 ±179	321.0 ± 171	0.746
***Protein (g/day)***	96.7 ± 59	85.4 ± 45	0.071
***Fat (g/day)***	100.0 ± 64	92.6 ± 42	0.263
***Fiber(g/day)***	22.3 ± 14	20.2 ± 11	0.180

^***1***^SD = standard deviation.

## Discussion

This study shows a significant improvement in 10 years coronary heart disease risk, based on Framingham risk score.To the best of our knowledge, there was no similar study among cardiovascular patients to assess the effect of Ramadan fasting on cardiovascular risks. Most of the studies evaluated changes of some cardiovascular risk factors among metabolic syndrome, diabetic, hyperlipidemic patients and or healthy adults.

There was a significant higher HDL-c and lower cholesterol, triglycerides, LDL-c, VLDL-c levels, cholesterol/HDL and LDL/HDL ratios after Ramadan in the present study. In Helsinki Heart Study a 10% reduction in TC, LDL-c (11%), 35% decrease in triglycerides and 11% increase in HDL-c after the intervention with pharmacological agent was associated with a 34% reduction in cardiovascular event [28]. A 2 unit increment of TC/HDL adds an excess of 68% to CHD risk [27,29]. Our study demonstrates significant improvement (−0.6) in TC/HDL and other lipids profile after Ramadan fasting which might be associated with a nearly 20% reduction in the risk of CHD.

A reduction in cholesterol [30,31], and triglycerides levels [30-32], has been reported in previous studies; however, The result of some other reports showed no significant changes in cholesterol [1,12,32-34] and triglycerides [1,2,33,34]. In a study in Kuwait on sixteen sedentary healthy adult males, there was no significant change in TG level after Ramadan [33]. This discrepancy was possibly due to small sample size or different Ramadan diet pattern in Kuwait study which includes more sweetened foods. Also, there are some reports about increased cholesterol [10,35] and triglycerides [10] after Ramadan fasting. There is a tendency for higher simple carbohydrate consumption during Ramadan [36].

Improvement in HDL-c has been shown in somestudies after fasting in Ramadan [2,30,31,34], while some other studies reported no significant change [1,32] or decrease [9,10] in HDL-c after Ramadan.

Significant decline in LDL-c has been reported in some studies after Ramadan fasting[2,30,31]. Also, no significant change [1,12,32,34] or significant increase [9,10,35] of LDL-c has been reported in other investigations.

It seems that the effect of Ramadan fasting on serum lipid levels may be closely related to the dietary habits and other life style changes [2,7].

There were significant decrease in systolic blood pressure and no significant change in diastolic blood pressures after Ramadan fasting in the present study. However, there are reports about significant decrease [1], no significant change [9,20,30,35] and significant increase [33] in systolic blood pressure.

There was no significant change in FBS, fasting insulin and insulin-resistance (HOMA-IR) after Ramadan fasting in the present study. Some studies showed no significant changes in the serum level of glucose [1,33,35]. While, the others reported higher [9] or lower [2,10,32] FBS after Ramadan fasting. These controversies may be explained by different food habits, amount of calorie intake [30], the number of fasting days, period of daily fasting, time of sampling, genetic tendency and daily activity in different reports. There is a report about significant increase in insulin sensitivity (1/HOMA IR) in subjects with the metabolic syndrome [8] and significant decrease in insulin and insulin resistance among men patients with type II diabetes after Ramadan fasting [35]. The lack of improvement in HOMA-IR or FBS despite reduction in body weight may be due to decrease in total body water content instead of body fat content after Ramadan fasting.

The value of hcy level did not change significantly after Ramadan in the present study. Although, Aksungar's study has been reported significant decrease in hcy after Ramadan fasting in 40 healthy volunteers [12].

Body weight and BMI decreased significantly in the present study (−1.4 kg and −0.7 kg/m2 respectively, *P <*0.001). Most reports are similar to these results [31,32]. Although, there are some reports about non-significant changes in body weight [37] and BMI [37,38] after Ramadan fasting.

In another study, no change was seen concerning anthropometric parameters [7].

### Limitations

There was no control group in this study. Pre Ramadan sampling in Ramadan was performed in the morning after an overnight fasting, while post Ramadan blood sampling was performed in the afternoon, after at least 10 h fasting during day time. This difference in the time of blood sampling was inevitable to have at least 10 h fasting. As the circadian rhythms of nutrition-related biological variables shows some degree of changes during Ramadan [39], there might be concerns about amount of daily activities or circadian rhythm of hormones like cortisol which might affect FBS levels. In the present study, we had only two measurements during the study, possibly evaluation in the middle of the month and after a time after Ramadan would reveal more details and shows us the trend of changes. Level of physical activity of the study population was not measured in this study. FFQ was completed for a control group not our subjects as this study was part of the bigger study that food data were completed as a separate study for a 500 volunteers to assess food pattern changes in Ramadan.

A multi-center study with larger sample size and other habitual changes controlled with matched control group is warranted.

## Conclusion

This study shows a significant improvement in10 years coronary heart disease risk score and other cardiovascular risk factors such as weight, BMI and waist circumference in subjects with a previous history of cardiovascular disease after an average of 26 d fasting during month of Ramadan. Result from this study suggested that Ramadan fasting may be useful to improve CVD risk factors.

## Competing interests

None of the authors had a personal or financial conflict of interest.

## Authors’ contributions

MN, AN, MAN: were responsible for study concept and design, MAN, MMR, MMF, SA, MS, SAM, MM SSS and NS: contributed for the design and conduct of the study, acquired the data, analyzed and interpreted data, performed statistical analysis and contributed to the writing manuscript. All authors read and approved the submitted manuscript.
